# Pharmacogenomic overlap between antidepressant treatment response in major depression & antidepressant associated treatment emergent mania in bipolar disorder

**DOI:** 10.1038/s41398-024-02798-y

**Published:** 2024-02-13

**Authors:** Nicolas A. Nuñez, Brandon J. Coombes, Lindsay Melhuish Beaupre, Aysegul Ozerdem, Manuel Gardea Resendez, Francisco Romo-Nava, David J. Bond, Marin Veldic, Balwinder Singh, Katherine M. Moore, Hannah K. Betcher, Simon Kung, Miguel L. Prieto, Manuel Fuentes, Mete Ercis, Alessandro Miola, Jorge A. Sanchez Ruiz, Gregory Jenkins, Anthony Batzler, Jonathan G. Leung, Alfredo Cuellar-Barboza, Susannah J. Tye, Susan L. McElroy, Joanna M. Biernacka, Mark A. Frye

**Affiliations:** 1 Department of Psychiatry & Psychology, Mayo Clinic, Rochester, MN USA; 2 Department of Quantitative Health Sciences, Mayo Clinic, Rochester, MN USA; 3 Department of Psychiatry, Universidad Autónoma de Nuevo León, Monterrey, México; 4 Lindner Center of HOPE/University of Cincinnati, Cincinnati, OH USA; 5 Department of Psychiatry & Behavioral Sciences, Johns Hopkins University, Baltimore, MD USA; 6 Department of Psychiatry, Faculty of Medicine, Universidad de Los Andes, Santiago, Chile; 7 Department of Pharmacy, Mayo Clinic, Rochester, MN USA; 8 Queensland Brain Institute, The University of Queensland, St. Lucia, QLD Australia

**Keywords:** Diseases, Bipolar disorder

## Abstract

There is increasing interest in individualizing treatment selection for more than 25 regulatory approved treatments for major depressive disorder (MDD). Despite an inconclusive efficacy evidence base, antidepressants (ADs) are prescribed for the depressive phase of bipolar disorder (BD) with oftentimes, an inadequate treatment response and or clinical concern for mood destabilization. This study explored the relationship between antidepressant response in MDD and antidepressant-associated treatment emergent mania (TEM) in BD. We conducted a genome-wide association study (GWAS) and polygenic score analysis of TEM and tested its association in a subset of BD-type I patients treated with SSRIs or SNRIs. Our results did not identify any genome-wide significant variants although, we found that a higher polygenic score (PGS) for antidepressant response in MDD was associated with higher odds of TEM in BD. Future studies with larger transdiagnostic depressed cohorts treated with antidepressants are encouraged to identify a neurobiological mechanism associated with a spectrum of depression improvement from response to emergent mania.

## Introduction

While there is a substantial evidence base for antidepressants (ADs) in major depressive disorder (MDD), the therapeutic benefit in bipolar disorder (BD) is far from conclusive [1]. The use of ADs, particularly in BD type I, remains controversial mainly due to a lack of consistent evidence for efficacy and potential concern for mood destabilization [2]. Data from the Systematic Treatment Enhancement Program for Bipolar Disorder (STEP-BD) showed that up to 40% of participants self-reported manic or hypomanic symptoms with ADs [3]. As recent data has suggested an increase in prescription rates of AD monotherapy in BD [4], there is an urgent need to identify clinical correlates or biomarkers of treatment non-response and/or adverse drug-related events, especially AD associated treatment-emergent mania (TEM) [5].

Genome-wide association studies (GWAS) have identified many genetic variants associated with BD [6] and a potential overlap in the biological pathways with MDD [7]; however, in many cases sample sizes may still remain too small to be able to detect significant variants associated with disease or treatment response. Additionally, there has been increasing evidence that would suggest a genetic overlap between MDD, BD and schizophrenia symptoms [8]. Polygenic scores (PGS) have become frequently used to examine how an individual’s genetic loading, computed from a combination of genetic variants, could predict risk to disease or clinical outcomes [9]. Specifically, a PGS is a weighted sum of a person’s alleles, creating a metric score used for risk quantification [10], diagnosis [11], or treatment response prediction [12, 13]. For example, studies focusing on treatment response have shown that a high genetic loading for schizophrenia or MDD in people with BD will decrease their response to lithium [14, 15].

In this study, our aim was to identify genetic factors associated with AD associated TEM in BD individuals and to explore a potential genetic overlap of AD response in MDD and TEM in BD.

## Methods

### Study population

Clinical and genomic data of adult patients with a BD diagnosis, prior AD treatment, and assessment of history of TEM were extracted from the Mayo Clinic Bipolar Biobank. The Biobank was established with the aim of building a repository that would facilitate studies on disease risk and pharmacogenomic treatment outcomes [16]. Enrollment sites included: Mayo Clinic (Rochester, Minnesota), Lindner Center of HOPE/University of Cincinnati College of Medicine (Cincinnati, Ohio), University of Minnesota (Minneapolis, Minnesota), Universidad des Los Andres (Santiago, Chile) and Universidad Autonoma de Nuevo Leon (Monterrey, Mexico). Each of the study sites received approval by their institutional review boards and every participant provided written informed consent at the time of inclusion into the study. Details of the primary study are described elsewhere [16]. A TEM case was defined by fulfilling DSM-IV criteria for a manic or hypomanic episode within 8 weeks of starting an AD or increasing its dose, while similar exposure to an AD with no TEM was considered as a control. ADs were grouped into selective serotonin reuptake inhibitors (SSRIs), serotonin and norepinephrine reuptake inhibitors (SNRIs), serotonin antagonist and reuptake inhibitors (SARIs – nefazodone trazadone), monoamine oxidase inhibitors (MAOIs), norepinephrine–dopamine reuptake inhibitor (NDRIs – bupropion), and tricyclic antidepressants (TCAs). For exploratory analyses, we restricted the cases to those who had history of TEM on specific AD classes. For these class-specific definitions, controls were considered those with adequate (≥8 weeks) exposure to the specific AD class and no history of TEM. A participant meeting criterion for TEM may have had multiple AD exposures and thus TEM history was documented for specific ADs.

### Genotyping and imputation for overall sample

Genotyping of the biobank sample was performed in three batches using different genomic platforms: (1) HumanOmniExpress-12v1-1; (2) GSA-24v2 and (3) Genotyping-by-Sequencing (GxS) performed by the Regeneron Genetics Center. Each batch was processed through the Mayo Clinic genotype quality control (QC) pipeline. In this QC pipeline, single nucleotide polymorphisms (SNPs) were excluded using filters for call rate (<95%), minor allele frequency (<0.5%), and Hardy-Weinberg Equilibrium (*p* < 1e-6). SNPs with a minor allele frequency less than 0.01 were also excluded Individuals were excluded for excessive missing genotypes (>5%), sex discrepancies, or abnormal heterozygosity (< 70% on multiple chromosomes). Each batch was then imputed using the TOPMed Imputation Server [17] and finally all three batches were merged. Relatedness within and across batches was estimated using KING-Robust [18]. For each pair with an estimated 2nd degree or higher relatedness, we preferentially kept those from the first batch, then those from the second batch and finally, those from the third batch. An individual was removed at random for related pairs within the same batch. Ancestry was estimated using ADMIXTURE [19]. Principal components (PCs) of ancestry were estimated using FlashPCA2 [20].

### Polygenic score for antidepressant response

The PGS for response to ADs was estimated using summary statistics from the largest GWAS of AD response in MDD (*N* = 5218) [21] with LDpred2 with auto-selection of tuning parameters [22]. The PGS was standardized to have standard deviation equal to one and a mean of zero. The GWAS of AD response in MDD included only individuals of European ancestries. Thus, because PGS can be biased if estimated in a target sample of different ancestry from the training ancestry, we restricted our analysis to include only samples of European ancestry. We excluded samples with a predicted probability of European ancestry of less than 80%. We also excluded subjects from Mexico and Chile due to the known admixed populations in those countries [23]. In total, this accounted for *N* = 101 patients being removed from the analysis leaving N = 861 patients of European ancestry with an assessment of TEM included in the analyses.

### Data analysis

We first conducted a GWAS of TEM in BD using logistic regression to test for associations between each SNP with TEM while adjusting for the first PC and the GWAS batch. For the PGS analysis, logistic regression was used to examine the association between the AD response PGS and TEM in the BD sample adjusting for the first PC, GWAS batch, and BD type because BD-I has been associated with higher risk of TEM [24]. Given the higher risk of TEM within BD- I, we further explored how this PGS predicted TEM in a subset of patients with BD-I. Finally, within this subset, we restricted our definition of TEM to ADs with different mechanisms of action (SSRI or SNRI) to examine how this affected the association between genetically predicted AD response and TEM.

## Results

A total of *N* = 313 (36%) patients had a history of TEM, while *N* = 548 (64%) patients had no history of TEM. When grouped by AD type, *N* = 221 (34%) and *N* = 139 (28%) had a history of TEM on SSRIs or SNRIs, respectively. Compared to patients without a history of TEM, patients with TEM were more likely to be female (69% vs. 60%; *p* = 0.011), have BD-I (78% vs. 69%; *p* = 0.0085) and, have a higher rate of history of serious suicide attempts (40% vs. 31%; *p* = 0.013). In addition, at study entry, patients with a history of TEM were less likely to be taking ADs (46% vs. 60%; *p* = 0.00013) and more likely to be currently using lamotrigine (39% vs. 25%; *p* = 0.00001). Table 1 summarizes participants’ demographic and clinical variables with or without a history of AD-associated TEM.

**Table 1:** Demographic and clinical variables in participants treated with antidepressants with or without a history of TEM.

	Total *N* = 861	No TEM *N* = 548	History of TEM *N* = 313	*P* value
Sex (female)	545 (63.3%)	329 (60.0%)	216 (69.0%)	0.011
Age, mean (SD)	43.1 (14.4)	44.8 (14.7)	40.2 (13.5)	8.5E-06
Race (White)	831 (96.52%)	529 (96.53%)	302 (96.49%)	0.37
Hispanic	10 (1.2%)	4 (0.8%)	6 (2.0%)	0.21
SNRI	293 (34.0%)	188 (34.3%)	105 (33.5%)	0.88
SSRI	689 (80.0%)	428 (78.1%)	261 (83.4%)	0.076
SCID Diagnosis				
Type I BD	622 (72.2%)	379 (69.2%)	243 (77.6%)	0.0085
Type II BD	227 (26.4%)	158 (28.8%)	69 (22.0%)	
Schizoaffective	12 (1.4%)	11 (2.0%)	1 (0.3%)	
History of Psychosis	356 (41.4%)	215 (39.2%)	141 (45.0%)	0.11
History of Psychosis during mania	275 (32.4%)	165 (30.6%)	110 (35.4%)	0.18
History of serious suicide attempts	294 (34.1%)	170 (31.0%)	124 (39.6%)	0.013
Adult Attention Deficit Disorder	200 (23.2%)	122 (22.3%)	78 (24.9%)	0.42
Generalized Anxiety Disorder	438 (50.9%)	264 (48.2%)	174 (55.6%)	0.043
Obsessive Compulsive Disorder	141 (16.6%)	89 (16.4%)	52 (17.0%)	0.91
Substance Abuse	422 (49.0%)	267 (48.7%)	155 (49.5%)	0.87
Current Medication Use				
Antidepressants	474 (55.0%)	329 (60.0%)	145 (46.3%)	0.00013
Lithium	248 (28.8%)	157 (28.6%)	91 (29.1%)	0.96
Lamotrigine	259 (30.1%)	136 (24.8%)	123 (39.3%)	1.2E-05
Divalproex/Valproic Acid	135 (23.6%)	92 (23.6%)	43 (23.5%)	1
Antipsychotics	422 (49.0%)	252 (46.0%)	170 (54.3%)	0.023
Benzodiazepines	292 (33.9%)	172 (31.4%)	120 (38.3%)	0.046

The GWAS of TEM did not identify any genome-wide significant variants. The top locus was (rs12929564 G/A; OR = 1.67; *p* = 2e-6) in an intronic region of *RBFOX1*. In the PGS analysis, a higher PGS for antidepressant response in MDD was associated with higher odds of TEM in BD (OR = 1.16 per SD increase in the PGS; *p* = 0.047). The PGS was more strongly associated with TEM in the BD-I sample (*N* = 622; OR = 1.27; *p* = 0.011) and was similar in BD-I individuals treated with SSRIs (*N* = 462; OR = 1.31; *p* = 0.016) or SNRIs (*N* = 352; OR = 1.32; *p* = 0.044). Conversely, the PGS association was not significant for the subset of BD-type II participants (*N* = 69; OR = 1.03; *p* = 0.811).

## Discussion

This study extends previous work examining clinical and genetic factors of antidepressant-associated TEM in BD. Our PGS analysis revealed a genetically predicted better AD response in MDD associated with higher odds of TEM in BD, particularly for BD-I.

Importantly, this study is the first to suggest that biological mechanisms underpinning AD response in MDD directly overlaps with those conferring the adverse event of TEM in BD or that depression improvement may represent a spectrum from response, remission, to emergent mania. Similarly, recent studies have underscored the value in the utilization of PGS in examining treatment resistance in MDD, and its overlap association with other psychiatric disorders such as schizophrenia [25] and therapeutic response to medications such as lithium [26]. Our PGS results did not identify a significant association with BD-II disorder, which mirrors clinical practice where ADs monotherapy has suggested relative efficacy and safety [24, 27]. Our PGS BD-I signal aligns with controlled clinical studies and current meta-analyses suggesting that the BD-I subtype has greater clinical and now also, genomic risk of TEM, despite higher rates of antimanic mood stabilization.

Similar to recent publications [27, 28], our GWAS did not identify any genome-wide significant variants. The top locus in our GWAS was an intronic variant in RNA binding fox-1 homolog 1 (*RBFOX1*), a gene highly expressed in the brain. *RBFOX1* regulates the expression of large genetic networks during neurodevelopment and was one of the top genes identified in the largest GWAS of mood disorders [29]. Importantly, RBFOX1 has been shown to critically regulate the expression of TrkB neurotrophin receptors to impact molecular signaling cascades conferring synaptic plasticity [30], and well-established to underpin mechanisms of AD response [31, 32].

Numerous hypotheses have highlighted the complexity surrounding ADs response and the emergence of side effects. Mechanistically, the monoaminergic hypothesis of AD response fails to fully address biological adaptations such as the AD-associated delayed therapeutic onset and neuroplasticity. Neurotrophic mechanisms dependent on brain-derived neurotrophic factor (BDNF) and mammalian target of rapamycin (mTOR) signaling promoting synaptic plasticity and activation of Wnt/ β-catenin signaling pathway have been proposed as an overlapping biological mechanism of action of ADs [31, 33–36]. Furthermore, the importance of mitochondrial energetics in the pathophysiology of BD is increasingly recognized [37, 38]. Interestingly, mitochondrial energetics may also play a role in AD associated TEM as recent data reported a significant increase in TEM rates in patients who were exposed to ADs that increase mitochondrial energetics [39], which would exert a downstream effect in the mTOR and Wnt/ β-catenin signaling pathways [35]. Importantly, the mTOR/Wnt/β-catenin signaling/pathway and mitochondrial energetics are mechanisms to further investigate in the spectrum of depression improvement from response to emergent mania aligning with the available genetic data.

We should consider several limitations of our study, most notably a negative GWAS result, likely because our study was underpowered due to the small sample size. This can be addressed by expanding to larger sample sizes in future GWAS and PGS analyses [40]. However, AD associated TEM studies will likely always have this limitation due to the complexities of collecting information on this phenotype. Our second limitation is the retrospective study design as to define the TEM phenotype. In addition, we did not analyze data from patients on AD monotherapy separately, and we should be aware of the possibility of a natural switch due to course of illness [5]. Considering our first limitation regarding a small sample size, we only included patients on specific classes of ADs such as SSRIs (*N* = 689) and SNRIs (*N* = 293) in our analysis. Finally, our PRS analysis was restricted to patients of European ancestry, which may limit the generalizability of our findings to other populations.

Nevertheless, our study has several strengths such as the inclusion of a TEM cohort confirmed by a thorough clinician review, not limiting only to self-report symptoms, with a narrow “window” of AD exposure. This study replicates previously established clinical risk factors for TEM (i.e., BD-I) [41] and would also be in accordance with previous registry data highlighting greatest risk within first three months of AD exposure [42].

In summary, our study extends previous literature on the clinical features and associations with AD associated TEM and supports the trans-diagnostic examination of the genetic overlap of treatment response across affective disorders (MDD and BD). Our findings suggest similar underlying genetic mechanisms between AD response in MDD and AD associated TEM in BD. Future work should focus on building larger samples examining AD mechanism of action and specific genetic variants which may provide a better understanding about the relationship between AD usage and TEM in BD.

## Data Availability

Data supporting the findings of this study are available from the corresponding author.
